# The Insect Pathogen *Serratia marcescens* Db10 Uses a Hybrid Non-Ribosomal Peptide Synthetase-Polyketide Synthase to Produce the Antibiotic Althiomycin

**DOI:** 10.1371/journal.pone.0044673

**Published:** 2012-09-18

**Authors:** Amy J. Gerc, Lijiang Song, Gregory L. Challis, Nicola R. Stanley-Wall, Sarah J. Coulthurst

**Affiliations:** 1 Division of Molecular Microbiology, College of Life Sciences, University of Dundee, Dundee, United Kingdom; 2 Department of Chemistry, University of Warwick, Coventry, United Kingdom; Vrije Universiteit Brussel, Belgium

## Abstract

There is a continuing need to discover new bioactive natural products, such as antibiotics, in genetically-amenable micro-organisms. We observed that the enteric insect pathogen, *Serratia marcescens* Db10, produced a diffusible compound that inhibited the growth of *Bacillis subtilis* and *Staphyloccocus aureus*. Mapping the genetic locus required for this activity revealed a putative natural product biosynthetic gene cluster, further defined to a six-gene operon named *alb1–alb6*. Bioinformatic analysis of the proteins encoded by *alb1–6* predicted a hybrid non-ribosomal peptide synthetase-polyketide synthase (NRPS-PKS) assembly line (Alb4/5/6), tailoring enzymes (Alb2/3) and an export/resistance protein (Alb1), and suggested that the machinery assembled althiomycin or a related molecule. Althiomycin is a ribosome-inhibiting antibiotic whose biosynthetic machinery had been elusive for decades. Chromatographic and spectroscopic analyses confirmed that wild type *S. marcescens* produced althiomycin and that production was eliminated on disruption of the *alb* gene cluster. Construction of mutants with in-frame deletions of specific *alb* genes demonstrated that Alb2–Alb5 were essential for althiomycin production, whereas Alb6 was required for maximal production of the antibiotic. A phosphopantetheinyl transferase enzyme required for althiomycin biosynthesis was also identified. Expression of Alb1, a predicted major facilitator superfamily efflux pump, conferred althiomycin resistance on another, sensitive, strain of *S. marcescens*. This is the first report of althiomycin production outside of the *Myxobacteria* or *Streptomyces* and paves the way for future exploitation of the biosynthetic machinery, since *S. marcescens* represents a convenient and tractable producing organism.

## Introduction

As the reported number of antibiotic resistant organisms continues to increase, the number of new antibiotics coming into use has dramatically declined over the past 20 years [1]. The majority of antibiotics that are in use within the clinic and in agriculture are derivatives of natural products and micro-organisms remain an essential source of potential drugs [2]. This has led to a surge in the mining of genomes and analysis of unique environmental niches in the search for novel antimicrobial compounds [3], [4]. In bacteria and fungi, many clinically relevant secondary metabolites (including antibiotics) are biosynthesized by non-ribosomal peptide synthetase (NRPS) enzymes, polyketide synthase (PKS) enzymes, or hybrids thereof. Examples include the clinically-relevant anticancer agents bleomycin A_2_ and ixempra (epothilone derivative), the anti-MRSA antibiotic dalfopristin (pristinamycin IIA/virginiamycin M1 derivative), and the pathogenicity-conferring siderophore yersiniabactin. The soil-dwelling bacteria *Streptomyces* and *Myxococcus* are particularly well known and prolific producers of such compounds [5], [6].

NRPS and PKS systems are large enzymes comprised of multiple modules. Both NRPS and PKS systems function in a very similar manner with each module within a system being responsible for the incorporation of a specific building block into the final product in an ‘assembly-line’ fashion [7]. NRPS systems incorporate proteinogenic and non-proteinogenic amino acids, as well as other types of carboxylic acid, whereas PKS systems generally utilise acyl-coenzyme A thioesters [8], [9], [10]. Each NRPS and PKS module contains a carrier protein domain that serves as the point of attachment for the growing peptide or polyketide chain via a phosphopantetheinyl “arm”. Phosphopantetheinyl transferase (PPTase) enzymes catalyse the addition of this 4′-phosphopantetheine (PPT) group to convert the carrier protein from an inactive to active state [11], [12]. Within each module responsible for the utilisation of a particular amino acid or acyl-CoA thioester, optional domains that modify the incorporated unit may also be found. Examples of these include epimerization, oxidase and N-methyltransferase domains in NRPSs, and ketoreductase, dehydratase, enoylreductase and C-methyltransferase domains in PKSs [13]. Trans-acting tailoring enzymes may also function to modify the product during or after peptide/polyketide chain assembly on NRPS/PKS multienzymes [8].

Althiomycin is a broad-spectrum antibiotic first isolated from *Streptomyces althioticus* in 1957 [14]. It is a heavily modified pentapeptide that inhibits protein biosynthesis by blocking the action of the peptidyl transferase [15], [16]. The X-ray crystal structure of the molecule was solved in 1974 [17] and the total chemical synthesis of althiomycin and analogues has been achieved (albeit with low efficiency) [18], [19]. However, the biosynthesis of althiomycin remained unexplored. The potential of althiomycin as a therapeutic agent is unclear. Results indicate the drug shows low cytotoxicity; however, there are conflicting reports over the extent to which it acts as a broad spectrum agent [15], [19]. To date, the difficulties associated with chemical synthesis of althiomycin have impeded further investigations into the effectiveness of althiomycin as a drug. Certain strains of the Gram-negative, soil-dwelling bacterium *Myxococcus xanthus* are known to produce althiomycin [20] and, while this manuscript was in preparation, a gene cluster that directs althiomycin biosynthesis in this organism was described [21].

*Serratia marcescens* is a Gram-negative bacterium belonging to the *Enterobacteriaceae*. *Serratia* strains produce various secondary metabolites, including several anti-microbial compounds [22]. The genetic tractability of *Serratia* has allowed a detailed dissection of how these secondary metabolites, including the antibiotics carbapenem and prodigiosin, are biosynthesized [22], [23], [24]. *S. marcescens* strain Db10 is a model insect pathogen and is a non-pigmented strain of *Serratia* [25]. Here we report the unexpected discovery that althiomycin is produced by this organism, as a metabolic product of a previously unidentified biosynthetic gene cluster. We observed that *S. marcescens* Db10 produced a diffusible metabolite able to inhibit the growth of *Bacillus subtilis*. A cluster of six genes encoding a hybrid NRPS-PKS assembly line, two tailoring enzymes and a putative self-resistance protein were identified as responsible for production of the antimicrobial. Additionally, a PPTase enzyme required for althiomycin biosynthesis was identified. Sequence analysis of the hybrid NRPS-PKS predicted that it assembled althiomycin (or a closely related molecule). Metabolite analysis of defined mutants confirmed this hypothesis, allowing a plausible pathway for althiomycin biosynthesis to be proposed. Moreover the genetic organisation of the gene cluster and resistance-conferring nature of the first gene in the operon were confirmed. Thus *S. marcescens*, a genetically-amenable enteric bacterium, is an unusual producer of this potentially valuable antibiotic.

## Results and Discussion

### *S. marcescens* Db10 is able to inhibit growth of Gram-positive bacteria

**Figure 1:**
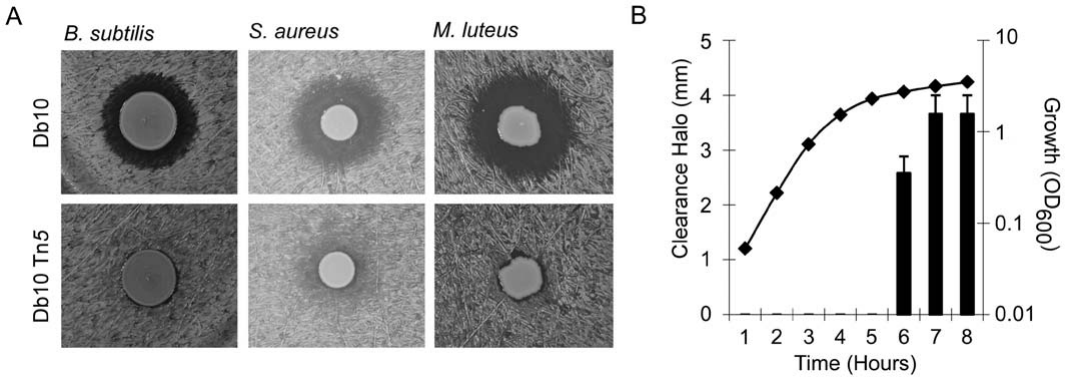
*S. marcescens* Db10 is able to inhibit growth of Gram-positive bacteria. **A.** Activity assays using *B. subtilis* NCIB3610, *S. aureus* 113 and *M. luteus* ATCC4698 as indicator strains, with *S. marcescens* Db10 or *S. marcescens* Db10 *SMA2290::*Tn*5* (NRS2992) as the producer strains. **B.** Culture supernatant assays (described in experimental procedures) indicate the diffusible molecule is produced by *S. marcescens* Db10 in stationary phase. Clearance halo sizes (radius of cleared area) are averages of three replicates; error bars represent standard error of the mean. A representative growth curve is shown for reference.

It was serendipitously discovered that *S. marcescens* Db10 produced a diffusible molecule capable of inhibiting the growth of the Gram-positive soil bacterium *Bacillus subtilis* (Figure 1A). To establish whether or not this effect was restricted to *B. subtilis*, the analysis was extended to include the Gram-positive human pathogen *Staphylococcus aureus* and Gram-positive human commensal *Micrococcus luteus*, revealing that these organisms are indeed also susceptible (Figure 1A). To establish the point in growth at which *S. marcescens* Db10 produced the molecule capable of inhibiting the growth of *B. subtilis*, the presence of this activity in the cell-free culture supernatant was assayed over 8 hours of growth. From this it was established that the activity was detectable in the stationary phase of growth, consistent with the diffusible compound being a secondary metabolite (Figure 1B). On the basis of these experiments it was concluded that *S. marcescens* Db10 was capable of inhibiting the growth of Gram-positive bacteria by biosynthesis of a diffusible compound with antimicrobial activity.

### Isolation of a mutant of *S. marcescens* Db10 unable to produce antimicrobial activity

**Figure 2:**
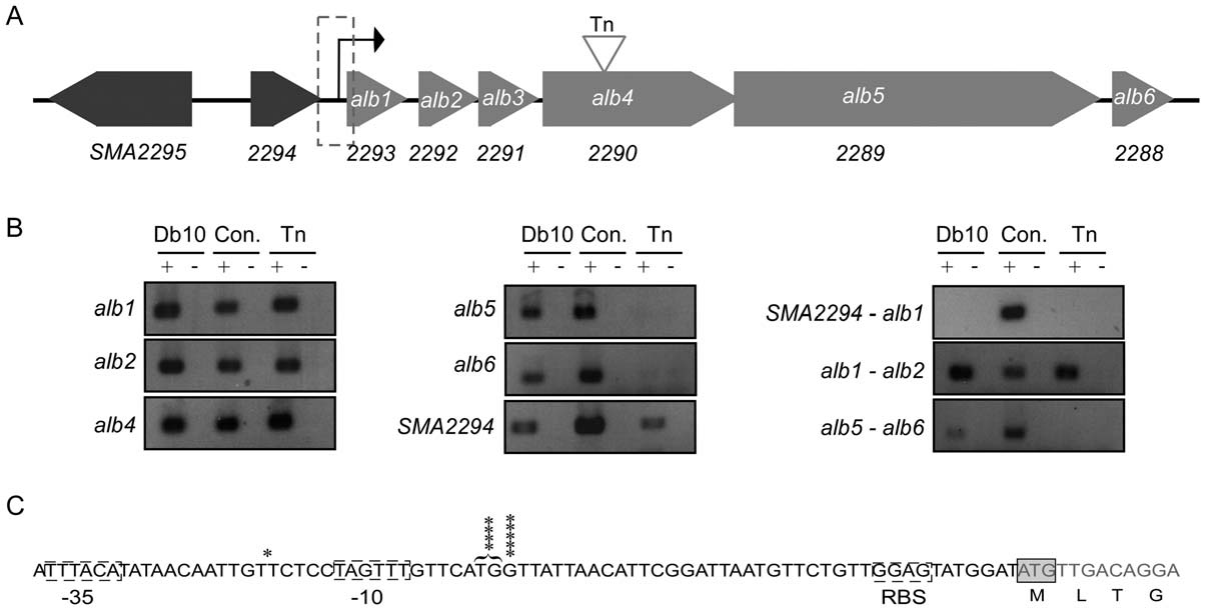
Transcriptional analysis of the *alb* operon. **A.** Map of the *alb* operon. Genes belonging to the *alb* operon are indicated as light grey arrows, genes that do not belong to the *alb* operon are indicated as dark grey arrows. Each gene is shown to scale. The region upstream of the *alb1* gene is highlighted by a broken box. The transcription start site is shown as a black arrow. The site of the transposon (Tn) insertion (at nucleotide 2745) in *alb4* is indicated with a grey triangle. **B.** RT-PCR analysis of the biosynthetic gene cluster. The template used in the PCR reaction is indicated above the gels as Db10 (wild type *S. marcescens* Db10 cDNA) or transposon (Tn*5* mutant cDNA). Reactions were performed in the presence (+) and absence (−) of reverse transcriptase. Con. represents *S. marcescens* Db10 genomic DNA as a positive control (+) and water as a negative control (−). The region of the chromosome amplified is indicated to the left of each gel. Primers were designed to amplify a product internal to a single gene, or spanning the intergenic region between two genes. Twenty five cycles of PCR amplification were used with the exception of *SMA2294* and *SMA2294-alb1* where 30 cycles were used. **C.** 5′ RACE analysis to identify the transcriptional start site of the *alb* operon. The region upstream of the *alb1* gene is shown and the methionine start codon is boxed. Asterisks indicate the number of times a particular base was identified as the transcriptional start site. Putative −10 and −35 regions are highlighted by a broken box. The −41 bp and −42 bp start sites are highlighted together as it is not possible to distinguish between these sites more specifically (see text).

Based on the hypothesis that *S. marcescens* Db10 synthesized and secreted an antimicrobial compound, random transposon mutagenesis was used to screen for mutant isolates of *S. marcescens* Db10 that could no longer inhibit the growth of *B. subtilis* and *S. aureus*. Six mutants were isolated exhibiting reduced killing activity towards both *B. subtilis* NCIB3610 and *S. aureus* 113 (e.g. Figure 1A). To ensure that disruption of the killing activity was associated with the transposon insertion, bacteriophage-mediated transduction was used to introduce one of the Tn5 insertions back into wild type *S. marcescens* Db10. The resulting strain (SJC13) displayed an identical phenotype to the original Tn5 isolate (not shown). Therefore we concluded that the lack of bioactivity was directly associated with the transposon insertion. The location of the transposon insertion in all six of the isolated transposon mutant strains was mapped, by interrogation of the publicly-available complete genome sequence of *S. marcescens* Db11 (Sanger Institute, UK). (Note that the sequenced strain, Db11, is a streptomycin-resistant derivative of the parental Db10 strain; the strains are otherwise identical and Db10 was used throughout this study.) All of the insertions were located within the coding region of *SMA2290* (Figure 2A). In each case the site of insertion was identical, indicating that the strains were most likely clonal isolates.

### Definition of the boundaries of the biosynthetic operon

Examination of the genetic context of *SMA2290* revealed it was the fifth of seven genes on the same strand (Figure 2A), and thus likely part of a biosynthetic operon. To define the 5′ boundary of the operon responsible for the biosynthesis of the potential antimicrobial compound, RT-PCR analysis was used. This was performed using cDNA synthesized from mRNA isolated from *S. marcescens* Db10 and *S. marcescens* Db10 *SMA2292*::Tn*5* (NRS2922). Primers designed to span the intergenic regions between 1) *SMA2294* and *SMA2293*; and 2) *SMA2293* and *SMA2292* were used to assess co-transcription. In addition, primers designed to amplify internal coding regions of *SMA2294*, *SMA2293*, *SMA2292*, *SMA2290*, *SMA2289* and *SMA2288* were used to confirm that the gene in question was expressed. We believe *SMA2293-SMA2288* (*alb1–alb6*) form a single operon since *alb1–alb2* are co-transcribed (Figure 2B), there is less than 5 bp between *alb2–3* and *alb4–5*, and translational coupling is observed between *alb3–alb4* (see below). Furthermore while there is a 146 bp intergenic gap between *alb5* and *alb6*, Alb6 is clearly needed for maximal althiomycin biosynthesis (see below) and insertion of the transposon in *alb4* disrupted transcription of both the downstream genes *alb5* and *alb6* (Figure 2B). We renamed genes *SMA2293-SMA2288* as *alb1–alb6* for **al**thiomycin **b**iosynthetic cluster.

The above findings indicated that *SMA2294* was not co-transcribed with *alb1* and that a promoter element should be present in the 305 bp gap between the stop codon of *SMA2294* and start codon of *alb1* to drive expression of *alb1–alb6*. To determine the location of the transcription start site, rapid amplification of cDNA 5′ ends (5′ RACE) was performed. The transcription start site was localised to −40 bp upstream and either −41 bp or −42 bp upstream of the *alb1* translation initiation codon (Figure 2C). It is not possible to distinguish between the −41 bp or −42 bp sites because the anchor primer that is needed for the 5′ RACE contains a series of thymine residues, which cannot be distinguished from the thymine residue at position −42 bp in the promoter region by sequencing (Figure 2C). Putative −10 and −35 binding sites for RNA polymerase were identified (Figure 2C). The −10 region (TAGTTT) shows a three out of six base pair match to the consensus sequence of TATAAT, whilst the −35 region (TTTACA) shows a five out of six base pair match to the consensus sequence of TTGACA. There is a 98 bp gap between the stop codon of *alb1* and the start codon of *alb2* but when 5′ RACE was applied to the region upstream from the *alb2* translation start site, no transcription initiation site could be identified (not shown). Taken together the data strongly indicate that *alb1–alb6* are co-transcribed, with *alb6* forming the final gene in the operon (Figure 2).

### Predictive sequence analysis of alb gene products

**Figure 3:**
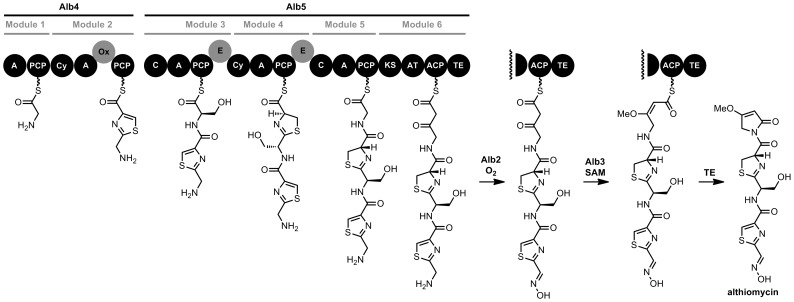
Proposed pathway for althiomycin biosynthesis in *S. marcescens* Db10. Alb4 and Alb5 form a hybrid NRPS-PKS assembly line, which consists of six modules as indicated. Domains within the NRPS-PKS are as follows. A: adenylation; PCP: peptidyl carrier protein; C: condensation; Cy: condensation/heterocyclisation; Ox: flavin-dependent oxidase; E: epimerisation; KS: ketosynthase; AT: acyl transferase; ACP: acyl carrier protein; TE: thioesterase. Alb6 (not shown) likely functions as an external ‘editing’ thioesterase. Alb3 is similar to known S-adenosylmethionine (SAM)-dependent methyltransferases and Alb2 is similar to known oxidases. The fact that no althiomycin-related compounds accumulate in *alb2*/*alb3* mutants suggests that N-oxidation and O-methylation take place in *trans* during chain assembly on the hybrid NRPS-PKS. However, the precise timing of the N-oxidation reaction is currently unclear.

The likely functions for each of the gene products in the *alb1–alb6* operon were deduced by sequence comparisons with proteins of known function. This analysis predicted that the operon encodes a three protein hybrid NRPS-PKS assembly line (*alb4, alb5* and *alb6*), associated tailoring enzymes (*alb2* and *alb3*) and an export and/or resistance protein (*alb1*). One of the genes encoding the hybrid NRPS-PKS multienzyme (*alb4*) was disrupted by the transposon insertion. The Alb4–5 NRPS-PKS multi-enzyme comprises six modules, the first two in Alb4 and the remainder in Alb5 (Figure 3). The first five modules are typical NRPS modules predicted to sequentially incorporate Gly, Cys, Ser, Cys and Gly residues [26], [27], with heterocyclisation of the two cysteine residues and oxidation to the thiazole of the thiazoline resulting from the first. The final module is a PKS module and is predicted to incorporate a malonyl unit [28]. The thioesterase domain at the end of Alb5 is hypothesized to catalyse release of the fully assembled chain (see below). The products of the *alb2* and *alb3* genes are predicted to act as ‘tailoring’ enzymes which further modify the NRPS-PKS-generated intermediate. Alb3 is predicted to be a SAM-dependent methyltransferase, whereas Alb2 is believed to be an *N*-oxygenase, exhibiting 41% sequence similarity to the aureothin biosynthetic enzyme AurF of *Streptomyces thioluteus* [29]. AurF is a diiron-dependent oxygenase that catalyses the 6-electron oxidation of 4-aminobenzoic acid to 4-nitrobenzoic acid. The product of *alb6* is predicted to function as a Type II thioesterase which likely represents an external ‘editing’ thioesterase. The *alb1* gene is predicted to encode a member of the major facilitator superfamily of membrane proteins, suggesting a possible role in self-resistance or efflux, as discussed further below.

Database searches for known compounds with structural similarity to the intermediates predicted to be assembled on the module 6 ACP domain suggested althiomycin (or a closely related molecule) as the likely metabolic product of the *alb* gene cluster. The conversion of the intermediate attached to the ACP domain of module 6 to althiomycin can be hypothesised to occur via Alb2-catalysed oxidation of the amino group in the intermediate to the corresponding oxime, followed by Alb3-mediated O-methylation. Subsequent TE-catalysed cyclisation of the resulting intermediate would afford althiomycin (Figure 3).

### Identification of the antimicrobial metabolite as althiomycin

**Figure 4:**
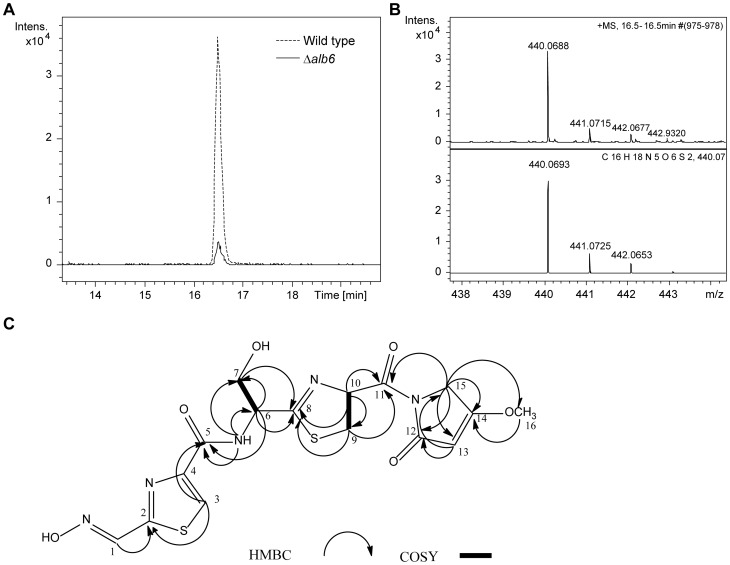
Spectroscopic analysis of althiomycin produced by *S. marcescens* Db10. **A**. Comparison of the extracted ion chromatogram at *m/z* 440.0600 for wild type *S. marcescens* Db10 (dashed line) and Db10 Δ*alb6* (solid line). **B**. Top panel: high resolution mass spectrum of althiomycin detected in culture supernatant of *S. marcescens* Db10. Bottom panel: simulated mass spectrum for the C_16_H_18_N_5_O_6_S_2_^+^ ion. **C**. Summary of COSY and HBMC NMR correlations observed for althiomycin isolated from *S. marcescens* Db10.

To determine whether the metabolic product of the *alb* gene cluster is althiomycin, ultra high resolution UHPLC-ESI-TOF-MS analysis of the culture supernatant from the wild-type strain and the *alb4* transposon mutant of *S. marcescens* Db10 was performed. This identified a compound present in the wild type, but lacking in the *alb4* transposon mutant, that yields an ion with the same molecular formula as protonated althiomycin (calculated *m/z* for C_16_H_18_N_5_O_6_S_2_^+^: 440.0688, observed: 440.0693, Figure 4A, Figure 4B and Figure S1). In order to unambiguously identify the metabolite as althiomycin, NMR spectroscopic analysis was performed on HPLC purified material (Figure 4C). Due to the rich medium used for production, some impurities were present. Nevertheless, careful inspection of ^1^H, COSY, HBMC and HSQC spectra confirmed that the compound is althiomycin (Table S3).

**Figure 5:**
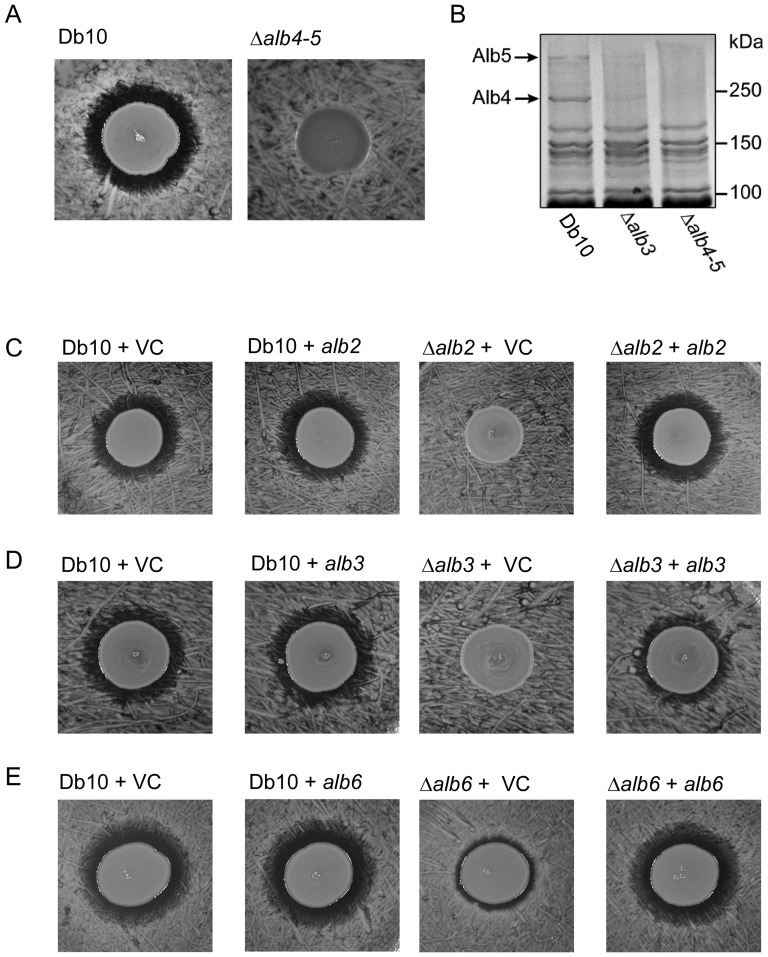
The tailoring enzymes are required for althiomycin production. **A**. Assay to assess antimicrobial activity of *S. marcescens* Db10 and the *alb4–5* mutant strain (SAN5) against *B. subtilis* 3610. **B.** Visualisation of proteins corresponding to the predicted size of Alb4 and Alb5. Total cellular protein from Db10 (wild type *S. marcescens* Db10), Δ*alb3* (SAN4) or Δ*alb4*–*5* (SAN5) mutants was isolated from cultures grown to stationary phase and separated by SDS-PAGE, followed by staining with Coomassie blue. **C–E.** Activity assays were performed using *Bacillus subtilis* NRS1473 as the indicator lawn. The producer strains used are indicated above as: Db10+VC (*S. marcescens* Db10 pSUPROM); Db10+*alb2* (*S. marcescens* Db10 pSAN2); Δ*alb2*+VC (SAN3 pSUPROM); Δ*alb2*+*alb2* (SAN3 pSAN2); Db10+*alb3* (*S. marcescens* Db10 pSAN3);Δ*alb3_51–262_*+VC (SAN88 pSUPROM); Δ*alb3_51–262_*+*alb3* (SAN88 pSAN3); Db10+*alb6* (*S. marcescens* Db10 pSAN38); Δ*alb6*+VC (SAN60 pSUPROM); Δ*alb6*+*alb6* (SAN60 pSAN38). ‘VC’ represents the empty vector control.

To further confirm that the lack of althiomycin production in the *alb4* Tn*5* mutant was due solely to disruption of the althiomycin biosynthetic gene cluster, an in-frame Δ*alb4–5* deletion strain (SAN5) was constructed. First, the ability of the Δ*alb4–5* deletion strain to inhibit the growth of *B. subtilis* was assessed. It was observed that the Δ*alb4–5* strain was unable to inhibit the growth of *B. subtilis* either when directly spotted onto an indicator lawn (Figure 5A) or when the culture supernatant was harvested and tested in the bioassay (not shown). Second, ultra high resolution UHPLC-ESI-TOF-MS analysis showed that althiomycin was lacking from the culture supernatant of the Δ*alb4–5* mutant (Figure S1). These findings unequivocally demonstrate that production of althiomycin is dependent on the *alb* gene cluster.

### The tailoring enzymes are required for althiomycin biosynthesis

Alb4 and Alb5, comprising the core NRPS-PKS machinery, are clearly required for althiomycin biosynthesis. To determine whether Alb2, Alb3 and Alb6 are also needed to produce althiomycin, in-frame deletions of *alb2, alb3* and *alb6* were constructed by allelic exchange. Similar to the Δ*alb4–5* mutant strain, the Δ*alb2* andΔ*alb3* strains no longer exhibited antimicrobial activity towards *B. subtilis* (Figure 5). The lack of althiomycin production was confirmed using ultra high resolution UHPLC-ESI-TOF-MS analyses (Figure S1). Interestingly, no potential intermediates in althiomycin biosynthesis could be detected in the culture supernatant of either mutant, suggesting that the modification reactions may occur on intermediates that are covalently attached to the assembly line. It is also possible that non-oxidised or non-methylated intermediates are not detected in the culture supernatant because they are unstable. However, it is unlikely that retention within the cell is responsible for our inability to detect potential intermediates because they were not detectable in cellular extracts either (data not shown). In contrast with the *S. marcescens* Db10 Δ*alb2* and Δ*alb3* mutant analysis, bioassays indicated that in the Δ*alb6* mutant strain a small amount of antimicrobial activity was retained (Figure 5E). Ultra high resolution UHPLC-ESI-TOF-MS analyses confirmed that althiomycin production in the Δ*alb6* mutant strain was roughly 10% that of the wild type Db10 strain (Figure 4A). These results indicate that *alb2* and *alb3* are essential for althiomycin production whereas Alb6 increases the efficiency of the process.

To confirm that the loss of althiomycin production in each of the mutant strains directly resulted from the in-frame deletion constructed, genetic complementation analysis was performed. To this end, medium-copy number plasmids carrying the complete *alb2, alb3* and *alb6* coding region*s* were introduced into the Δ*alb2*, Δ*alb3* and Δ*alb6* strains, respectively, and the bioassay was performed. The growth inhibition activity in all cases was restored (Figure 5). Interestingly, in the case of *alb3*, on construction of an almost-complete in-frame deletion, Δ*alb3* (SAN4), in which only a fusion of the first three and final ten amino acids remained, the althiomycin defect could not be complemented by expression of *alb3 in trans* (not shown). Given that there is only a 21 bp gap between *alb3* and *alb4*, we wondered if there might be translational coupling between these two genes. Supporting this idea, in the Δ*alb3* mutant, transcription of *alb4* and *alb5* was retained but SDS-PAGE analysis indicated that protein bands consistent with Alb4 (266.68 kDa) and Alb5 (597.17 kDa) were no longer easily detectable, compared with the wild type (Figure 5B; the identity of Alb4 as the primary constituent of the ∼250 kDa band in the wild type was confirmed by mass spectrometry; not shown). Generation of the Δ*alb3_51–262_* mutant (SAN88), missing 211 amino acids from the middle of the protein and thus preserving in-frame translation of the 3′ end of the gene, again abrogated althiomycin production, but this time the defect could be complemented be expressing *alb3 in trans* (Figure 5D). Furthermore, consistent with translational coupling between *alb3* and *alb4*, protein bands consistent with Alb4 and Alb5 were readily detected in theΔ*alb3_51–262_* mutant (SAN88) strain (not shown). This implies tight co-ordination between the production of Alb3 and Alb4–5, perhaps to ensure a 1∶1∶1 stoichiometry of Alb3:Alb4:Alb5 which would be consistent with O-methylation of a PKS-bound intermediate.

### Identification of a PPTase enzyme required for althiomycin biosynthesis

PPTase enzymes catalyse the transfer of a PPT group from coenzyme A to a conserved serine residue within the carrier protein domains of NRPS and PKS enzymes and this modification is essential for the activity of the enzyme [11]. The gene encoding the PPTase required for activation of an NRPS or PKS enzyme may or may not be encoded in the same region of the genome as the NRPS or PKS biosynthetic gene cluster [30]. The *alb1–alb6* gene cluster does not encode a PPTase enzyme; therefore we extended our search for the PPTase needed for althiomycin biosynthesis to the entire genome of *S. marcescens* Db10.

**Figure 6:**
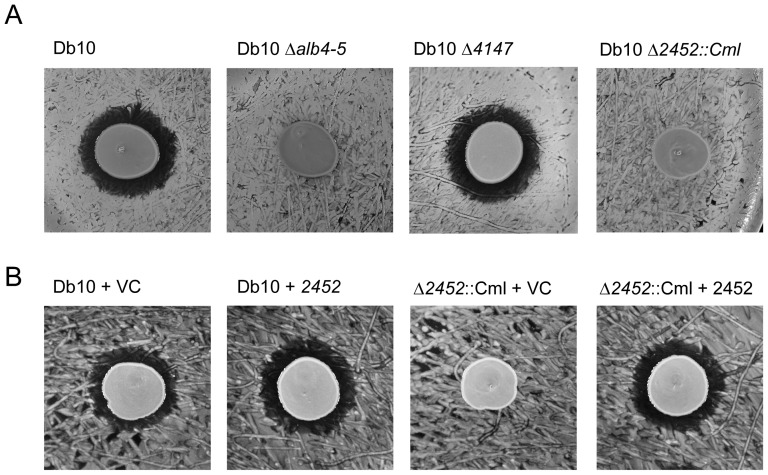
The PPTase encoded by *SMA2452* is required for althiomycin biosynthesis. **A.** Assay to assess the antimicrobial activity of *S. marcescens* Db10, the *alb4–5* mutant strain (SAN5), the Δ*4147* mutant strain (SAN96) and the Δ*2452*::Cml mutant strain (SAN112) against *B. subtilis* 3610. **B.** Activity assays were performed using *Bacillus subtilis* NRS1473 as the indicator lawn. The producer strains are indicated above as: Db10+VC (*S. marcescens* Db10 pSUPROM); Db10+2452 (*S. marcescens* Db10 pSAN46); Δ*2452*::Cml+VC (SAN112 pSUPROM); Δ*2452*::Cml+*2452* (SAN112 pSAN46). ‘VC’ represents the empty vector control.

PPTase enzymes are divided into three classes based on the type of carrier protein domain that they modify [31]. The Sfp class of PPTases generally activate the carrier protein domains of NRPS and PKS enzymes involved in secondary metabolism and are so-called after the surfactin PPTase, Sfp, of *B. subtilis* [32]. EntD of *Escherichia coli* also belongs to the Sfp class of PPTases and is required for enterobactin biosynthesis [12]. Two PPTases in *S. marcescens* Db10 were identified based on their similarity to Sfp of *B. subtilis* and EntD of *E. coli*; *SMA4147* and *SMA2452*, respectively (Figure S2). These represented likely candidates for the PPTase needed for althiomycin biosynthesis and deletion strains were constructed. Althiomycin production was assessed using the *B. subtilis* bioassay and by UHPLC-ESI-TOF-MS analysis. Deletion of *SMA4147* did not affect althiomycin production; in contrast, mutation of *SMA2452* abolished althiomycin production (Figure 6A and Figure S2). To confirm that the loss of althiomycin production in the *SMA2452* mutant strain was specific to this gene, complementation analysis was performed. To this end, a plasmid carrying the complete *SMA2452* coding region was introduced into the *SMA2452* mutant strain, and the bioassay was performed. The growth inhibition activity was restored (Figure 6B). It is therefore reasonable to conclude that *SMA2452* functions to phosphopantetheinylate the Alb4–5 multi-enzyme during althiomycin biosynthesis.

### Alb1 is required for resistance to althiomycin

**Figure 7:**
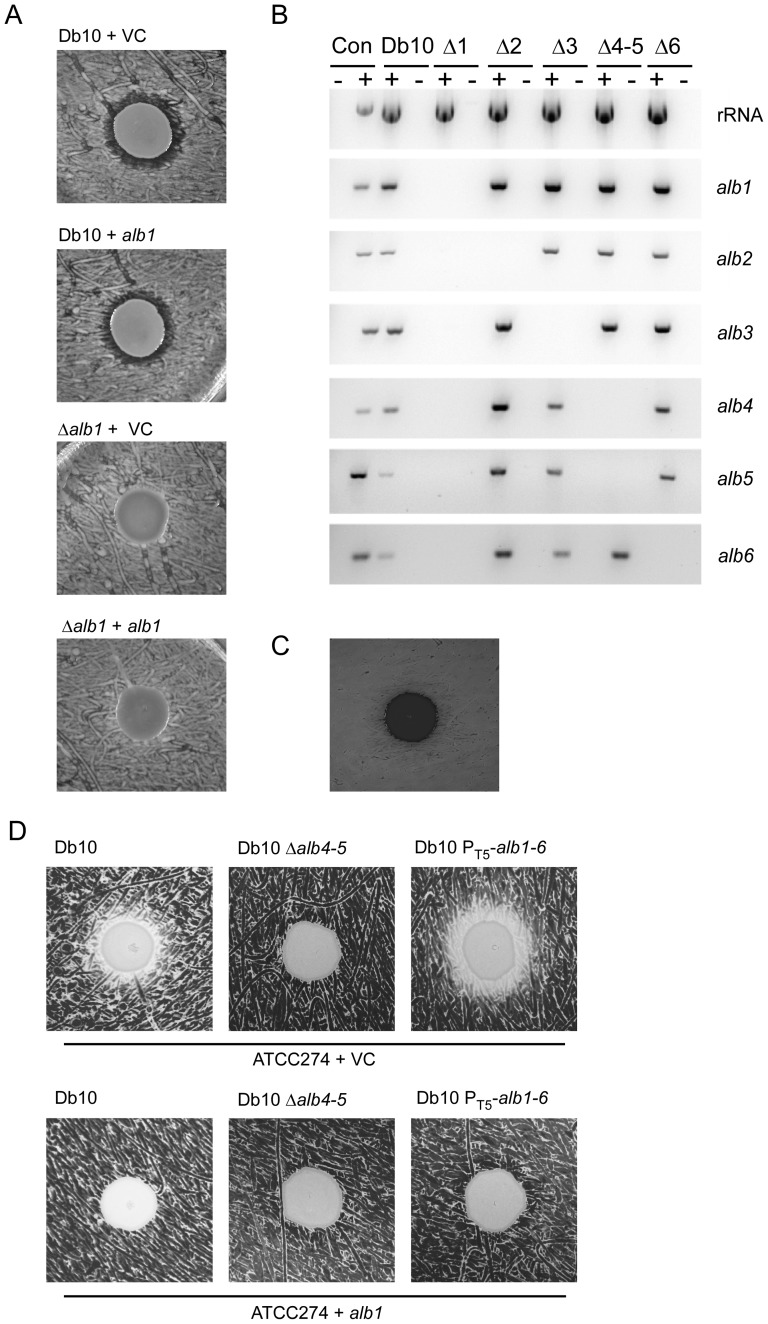
The *alb1* gene encodes an althiomycin resistance determinant. **A**. Activity assays using *B. subtilis* NRS1473 as the indicator lawn for the producer strains: Db10+VC (*S. marcescens* Db10 pSUPROM); Db10+*alb1* (*S. marcescens* Db10 pSAN1); Δ*alb1*+VC (SAN2 pSUPROM); Δ*alb1*+*alb1* (SAN2 pSAN1) **B.** RT-PCR analysis of *alb1*–*alb6* transcript levels in each of the *alb* mutant strains. The template used in the PCR reaction is indicated above the gels as: Db10 (wild type *S. marcescens* Db10 cDNA); Δ1 (SAN2(Δ*alb1*) cDNA); Δ2 (SAN2 (Δ*alb2*) cDNA); Δ3 (SAN4 (Δ*alb3*) cDNA); Δ4–5 (SAN5 (Δ*alb4–5*) cDNA); Δ6 (SAN60 (Δ*alb6*) cDNA). Reactions were performed in the presence (+) or absence (−) of reverse transcriptase. Con. represents *S. marcescens* Db10 genomic DNA as a positive control (+) and water as a negative control (−). The primer pairs used to amplify a product internal to a particular gene are indicated to the right of each gel. Twenty five cycles of PCR amplification were used. **C.** Activity assays using *B. subtilis* 3610 as the indicator lawn and *S. marcescens* ATCC274 as the producer strain. **D.** Activity assays using Db10 (*S. marcescens* Db10 pSUPROM), Db10 Δ*alb4–5* (SAN5 pSUPROM) and Db10 P_T5_-*alb1–6* (SAN100 pSUPROM) as the producer strain and using ATCC274+VC (*S. marcescens* 274 pSUPROM) and ATCC274+*alb1* (*S. marcescens* 274 pSAN1) as the indicator lawn. ‘VC’ represents the empty vector control.

Alb1 is predicted to be a member of the major facilitator superfamily of membrane proteins. In many cases these proteins act as export or efflux pumps [33]. In antibiotic producing organisms, resistance genes are commonly located within the antibiotic biosynthetic gene cluster [34]. We therefore hypothesised that Alb1 was required for self-resistance to althiomycin and/or export of althiomycin from the cell into the surrounding environment. We predicted that if *alb1* was essential for self-resistance, it might not be possible to make an althiomycin-producing *alb1* mutant due to lethality. Surprisingly, an *alb1* mutant was successfully constructed. However, this mutant (SAN2) did not produce extracellular althiomycin according to the *B. subtilis* activity assay (Figure 7A). In an attempt to complement the Δ*alb1* mutant phenotype, *alb1* was expressed *in trans* using exactly the same vector system as was used to successfully complement the *alb2*, Δ*alb3_51–262_* and *alb6* mutant phenotypes. However, no complementation could be observed (Figure 7A).

To investigate why genetic complementation of the *alb1* mutant was unsuccessful, the impact of deleting each of the *alb* genes on transcription within the *alb* operon was assessed by RT-PCR using the wild type *S. marcescens* Db10 and the Δ*alb1* (SAN2), Δ*alb2* (SAN3), Δ*alb3* (SAN4), Δ*alb4–5* (SAN5) and Δ*alb6* (SAN60) deletion strains. We observed that deletion of *alb2–6* had no effect on transcription of other genes in the *alb* gene cluster (Figure 7B). However, in the Δ*alb1* strain, we could only detect a very low level of transcription from each of the remaining *alb* genes (Figure 7B). Expression of *alb1 in trans* in the Δ*alb1* mutant was not sufficient to restore transcription of *alb2–6* to wild type levels, thus explaining why it was not possible to genetically complement the *alb1* mutant strain (not shown). If Alb1 is indeed required for resistance, these results explain why it was possible to construct a Δ*alb1* mutant, suggesting there may be an inbuilt survival mechanism contained within the althiomycin biosynthetic gene cluster that blocks production of the antibiotic in the absence of the putative resistance protein. Alternatively we cannot currently rule out the possibility that during the construction of *alb1* deletion strains, second site mutations blocking althiomycin biosynthesis are acquired, perhaps even in a self-protecting mutagenic ‘hot-spot’.

We predicted that if Alb1 was both necessary and sufficient to confer resistance to althiomycin, introduction of the *alb1* coding region into a bacterial strain that was sensitive to althiomycin should confer resistance. To this end, we first tested whether *S. marcescens* ATCC274, a prodigiosin-producing environmental isolate of *S. marcescens*, produced althiomycin. This was assessed using two methods: firstly *S. marcescens* ATCC274 was found to be unable to produce a zone of inhibition in the *B. subtilis* bioassay (Figure 7C). Secondly, PCR using primers specific to the *S. marcescens* Db10 *alb* gene cluster indicated the *alb* operon was most likely absent from *S. marcescens* ATCC274 (not shown). Consistent with *S. marcescens* ATCC274 not producing althiomycin, we also showed that it was sensitive to althiomycin produced by *S. marcescens* Db10 (Figure 7D), providing an ideal heterologous background to test the role of Alb1. The coding region for Alb1 was expressed in *S. marcescens* ATCC274 and resistance of the transformed strain to althiomycin was observed. Introduction of Alb1 into *S. marcescens* ATCC274 was indeed able to eliminate the antimicrobial effect of althiomycin, demonstrating that *alb1* does confer resistance to althiomycin (Figure 7D). Since the zone of growth inhibition surrounding the *S. marcescens* Db10 spot on the *S. marcescens* ATCC274 lawn was relatively small, we generated a strain of Db10 producing higher levels of althiomycin to further confirm this result. The native althiomycin promoter was replaced with the constitutive T5 promoter on the chromosome of *S. marcescens* Db10. This strain was found to produce increased levels of althiomycin (not shown). Using this strain as the althiomycin producer, we were able to clearly demonstrate that resistance to althiomycin was observed in the *S. marcescens* ATCC274 strain expressing Alb1 (Figure 7D).

### Comparison with althiomycin biosynthesis in Myxococcus

Concurrent with our independent, *de novo* identification, analysis and characterisation of the althiomycin biosynthetic gene cluster of *S. marcescens* Db10, a similar cluster directing althiomycin production was identified in the Gram-negative, soil dwelling bacterium, *Myxococcus xanthus* strain DK897 [21], following earlier reports of althiomycin production by this organism [20]. *S. marcescens* Db10 and *M. xanthus* are only distantly related, being members of the Gamma and Delta Proteobacteria, respectively. Therefore to determine the level of similarity between the two althiomycin biosynthetic gene clusters, a comparative bioinformatic analysis was performed (Table 1). The althiomycin biosynthetic gene clusters of *M. xanthus* and *S. marcescens* Db10 both contain six genes: *almA-F* and *alb1–6*, respectively. Five of the six gene products are clearly equivalent and the gene order is conserved, suggesting that the clusters are related. The Alb1–5 proteins share reasonable identity with the corresponding proteins in *M. xanthus* at the primary amino acid sequence level (Table 1). Our predictions for the role played by each of these proteins in althiomycin biosynthesis by *S. marcescens* Db10 are similar to those made for the corresponding proteins in *M. xanthus* [21]. Surprisingly however, Alb6 and AlmF show no detectable identity with each other at the primary amino acid sequence level. Alb6 is predicted to be a type II thioesterase, with an ‘editing’ or ‘proofreading’ function, required for the removal of misloaded building blocks or incorrect intermediates from the NRPS/PKS machinery [13]. This finding is corroborated by the finding that althiomycin production is reduced to ∼10% of wild type levels in the *alb6* mutant. By contrast, AlmF of *M. xanthus* is predicted to be a proline iminopeptidase (TIGR01250) and Cortina *et al.* (2011) prefer a model where AlmF catalyses the formation of the methyoxypyrrolinone ring of althiomycin following release from the NRPS-PKS enzyme. These authors do note, however, that this step could also be performed by the integrated (Type I) thioesterase domain at the end of AlmB (equivalent to that of Alb5). Alb6 and AlmF are both members of the α/β hydrolase superfamily of proteins (Pfam 12697) and consistent with this, similarities in the predicted protein secondary structures of these proteins can be seen (not shown). Additionally, minor differences exist in the organisation of the biosynthetic operons between the two organisms. In *M. xanthus*, *almB* and *almF* (equivalent to *alb5* and *alb6*) are predicted to be translationally coupled. In the *S. marcescens* Db10 *alb* gene cluster, *alb5* and *alb6* are separated by a 146 bp gap suggesting that translational coupling is unlikely.

**Table 1:** Comparative Analysis of the Althiomycin Biosynthetic Proteins.

*S. marcescens*	Length^a^	*M. xanthus*	Length^a^	% identity/similarity^b^	Proposed Function
Alb1	393	AlmE	446	56/72	Resistance/export
Alb2	318	AlmD	317	55/70	Fe/Mn-dependent N-oxygenase
Alb3	303	AlmC	302	55/69	O-Methyltransferase
Alb4	2376	AlmA	2396	44/59	NRPS
Alb5	5347	AlmB	5428	46/61	NRPS/PKS
Alb6	244	AlmF	322	None	Thioesterase

a The length of the protein is given as the number of amino acids.

b The percentage similarity and identity was obtained using pairwise protein-protein BLAST analysis across the entire length of the protein [41].

The identification of related althiomycin biosynthetic gene clusters in strains of two almost unrelated Gram-negative bacteria, together with the historic observation of althiomycin production by the actinomycete, *S. althioticus* [14] strongly suggests that *S. marcescens* Db10 has acquired the *alb* genes by horizontal gene transfer. However there is no obvious evidence for recent horizontal transfer of the *alb* genes in *S. marcescens* Db10; the G+C content of the cluster is not significantly distinct from that of the whole genome and nor are there any obvious remnants of mobile genetic elements visible nearby (not shown).

### Conclusions and implications

In this work we report the unexpected observation that a strain of *S. marcescens*, an enteric bacterium and an opportunistic pathogen, produces the broad-spectrum antibiotic althiomycin. Biosynthesis of althiomycin by *S. marcescens* Db10 is directed by a six-gene operon encoding a hybrid NRPS-PKS assembly line, essential tailoring enzymes and a cognate self-resistance protein. The operon does not encode a dedicated phosphopantetheinyl transferase (PPTase) enzyme, required for post-translational phosphopantetheinylation of the PCP/ACP domains within the NRPS-PKS [13]. However we have identified a PPTase required for althiomycin biosynthesis, the product of *SMA2452*. The PPTase required for althiomycin production in *M. xanthus* has not been reported.

This is the first report of althiomycin biosynthesis by an organism outside of the *Myxobacteria* or *Streptomyces*, both representing soil-dwellers well known for producing antibiotics. In contrast, *S. marcescens* is a close relative of enteric bacteria such as *Yersinia* and *E. coli* and an opportunistic pathogen. The production of althiomycin may confer a significant competitive advantage for the survival of *S. marcescens* Db10, since the antibiotic is readily produced under normal culture conditions despite the high energetic burden of biosynthesizing the extremely large NRPS-PKS proteins. Whether this advantage is particularly relevant to an insect-pathogenic lifestyle remains to be seen, but this is possible, since Db10 is an insect pathogen, unlike *S. marcescens* ATCC274 and other *Serratia* strains which do not elaborate althiomycin.

**Figure 8:**
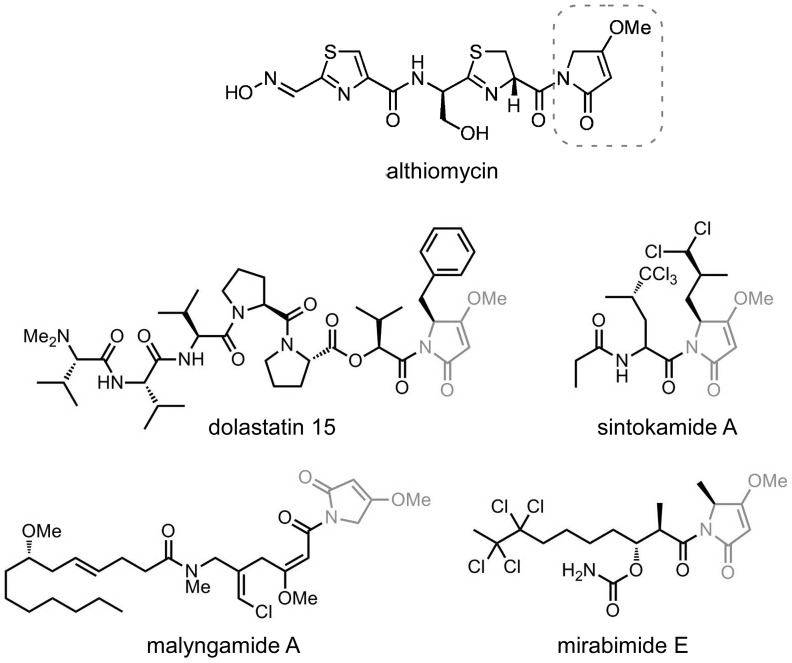
The 4-methoxy-3-pyrrolin-2-one moiety of althiomycin is shared by other bioactive natural products. The position of the 4-methoxy-3-pyrrolin-2-one moiety in althiomycin is highlighted by a broken box and this moiety is depicted in grey within the other molecules.

Althiomycin has attracted pharmaceutical interest as a potentially useful antibiotic, but progress has been curtailed by the lack of efficient chemical syntheses and availability of the biosynthetic genes/enzymes. Furthermore, of broader interest is the 4-methoxy-3-pyrrolin-2-one moiety of althiomycin (Figure 8). This is a key structural feature of numerous other bioactive natural products that are mostly of marine origin, including sintokamide A, malyngamide A, mirabimide E and the extremely potent anticancer agent dolastatin 15 (Figure 8). Nothing is known about the biosynthesis of these remarkable natural products [35], [36], [37], [38]. We hypothesise that they, like althiomycin, are the products of hybrid NRPS-PKS assembly lines and that the 4-methoxy-3-pyrrolin-2-one unit in these molecules is assembled by analogous enzymatic logic to that employed in the assembly of althiomycin (Figure 3). Thus, NRPS-mediated chain extension with glycine (in the case of malyngamide) or L-phenyalanine (in the case of dolastatin-15) followed by PKS-mediated chain extension with malonyl-CoA, O-methylation of the resulting beta-ketothioester and thioesterase-mediated cyclisation, equivalent to the action of Alb3 and the final two modules of Alb5, would yield the corresponding 4-methoxy-3-pyrrolin-2-ones.

Modules and domains of NRPS and PKS enzymes have proven amenable to ‘mix and match’ combinatorial approaches to generating novel bioactive molecules. Therefore the identification and characterisation of the althiomycin biosynthetic gene cluster in *S. marcescens*, which is a fast-growing and highly genetically tractable organism, offers an exciting “tool box” for the potential future exploitation of Alb proteins as a starting point for the generation and synthesis of useful and modified natural products, particularly those bearing the important 4-methoxy-3-pyrrolin-2-one pharmacophore. Furthermore, interesting questions remain regarding the biological and ecological significance of production of this antibiotic by a pathogenic organism, and as to how widespread this gene cluster is amongst diverse bacterial genera and lifestyles.

## Materials and Methods

### Strains and culture media

*B. subtilis* and *Escherichia coli* were routinely cultured in Luria-Bertani (LB) medium (1% Bacto tryptone, 0.5% Bacto yeast extract, 1% NaCl). *S. marcescens* and *Micrococcus luteus* were routinely cultured in low salt Luria-Bertani (LB) medium (1% Bacto tryptone, 0.5% Bacto yeast extract, 0.5% NaCl). S. *aureus* was routinely grown in Tryptic Soya Broth (TSB) (0.5% NaCl, 0.5% soytone, 1.5% tryptone). Growth media were solidified through addition of agar to 1.5%. When required, media were supplemented with antibiotics: ampicillin (Ap) 100 µg/ml; kanamycin (Kan) 50 µg/ml (*E. coli*) or 100 µg/ml (*S. marcescens* and *B. subtilis*); streptomycin (Sm) 100 µg/ml, chloramphenicol (Cml) 100 µg/ml and tetracycline (Tc) 10 µg/ml.

### Construction of strains and plasmids

*S. marcescens* chromosomal mutants with in-frame deletions or marked mutations in selected genes were constructed by marker (allelic) exchange using the suicide vector pKNG101, as described [39]. The T5 promoter was PCR-amplified from pQE-80 and used to replace the native chromosomal promoter by the same method. The primers used to amplify and clone the appropriate regions are detailed in Table S1 and the relevant plasmids are given in Table 2 and Table S2. For complementation of the *S. marcescens* Δ*alb2* (SAN3), Δ*alb3* (SAN88), Δ*alb6* (SAN60) and Δ*2452*::Cml mutants, the *alb2, alb3*, *alb6* or *SMA2452* coding region was amplified from genomic DNA of *S. marcescens* Db10. The PCR product was cloned into the vector pSUPROM using the restriction sites introduced into the primer sequence (Table S1). The integrity of the coding regions was confirmed by DNA sequencing. The resulting plasmids (Table 2 and Table S2) were introduced into *S. marcescens* Db10 strains by electroporation and selecting with kanamycin (100 µg/ml) [40].

**Table 2:** Selected Bacterial Strains and Plasmids Used in This Study.

Strain/Plasmid	Description	Reference^b^
Db10	*Serratia marcescens* Wild type	[25]
NRS2992	Db10 *SMA2290::*Tn*5*	This study
SAN2	Db10 (Δ*alb1*) in-frame	This study
SAN3	Db10 (Δ*alb2*) in-frame	This study
SAN4	Db10 (Δ*alb3*) in-frame	This study
SAN88	Db10 (Δ*alb3_51–262_*) in-frame	This study
SAN5	Db10 (Δ*alb4–5*) in-frame	This study
SAN60	Db10 (Δ*alb6*) in-frame	This study
SAN96	Db10 (Δ*SMA4147*) in-frame	This study
SAN112	Db10 (Δ*SMA2452*::Cml)	This study
SAN100	Db10 P_T5_-*alb1–6* (T5 promoter replacing native promoter upstream of *alb1*)	This study
ATCC274	*Serratia marcescens* Wild type	A.T.C.C.
**Plasmids**		
pSUPROM	Vector for constitutive expression of cloned genes under the control of the *E. coli* Tat promoter (Kan^R^)	[44]
pSAN1	*alb1* coding sequence in pSUPROM	This study
pSAN2	*alb2* coding sequence in pSUPROM	This study
pSAN3	*alb3* coding sequence in pSUPROM	This study
pSAN38	*alb6* coding sequence in pSUPROM	This study
pSAN46	*SMA2452* coding sequence in pSUPROM	This study

### Activity assays

The indicator strain was routinely grown at 37°C to early stationary phase and 100 µl of the culture was spread onto an agar plate. *S. marcescens* strains being tested for antibiotic production were grown to stationary phase at 30°C and 10 µl of culture diluted to an OD_600_ of 1 was spotted on the indicator lawn. To test the ability of the *alb2, alb3*, *alb6* or *SMA2452* complementation strains to produce althiomycin, *B. subtilis* strain NCIB3610 *sacA*::Phy-spac-*gfp* (Kan) (NRS1473) was used as the indicator and 100 µg/ml of kanamycin used to maintain replicative plasmids in *S. marcescens* strains. To test the ability of Alb1 to confer resistance to althiomycin in *S. marcescens* ATCC274, *S. marcescens* ATCC274 carrying pSUPROM or pSUPROM-*alb1* (pSAN1) was used as the indicator lawn and was grown at 30°C to early stationary phase before plating. 100 µg/ml of Kan was added to the agar plate. The plates were incubated (15 h) at 30°C prior to photography. Images were captured using a Leica MZ16 FA stereoscope using LAS software version 2.7.1.

To assess althiomycin production throughout growth, *S. marcescens* Db10 and SAN5 were grown in liquid culture. At hourly intervals the OD_600_ was measured and a sample was collected. The cells were removed and the supernatant was filter sterilized (0.2 µm). To prepare indicator lawns, a liquid culture of *B. subtilis* NCIB3610 was diluted 1 in 1000 into LB top agar (0.7% agar w/v) and poured onto an LB plate. 5 mm wells were bored and filled with the culture supernatant. Plates were incubated at 37°C for 6 hours, until the *B. subtilis* lawn was clearly visible and the halo was measured.

### Generation and mapping of *S. marcescens* Db10-Tn5 mutants

To obtain *S. marcescens* transposon mutants, *S. marcescens* Db10 was incubated in the presence of the transposon donor strain *E. coli* S17-1 λPir harbouring plasmid pUT-mini-Tn*5*l*acZ*1 [39]. Individual *S. marcescens* Db10 Tn*5* mutant colonies were patched onto an LB agar plate and an LB agar plate seeded with a lawn of *B. subtilis* strain NCIB3610. In total 2800 *S. marcescens* Db10 Tn*5* mutants were screened. Of these, 6 colonies that showed a decreased ability to kill *B. subtilis* NCIB3610 were isolated and purified. The location of the insertion was mapped using inverse PCR of *Taq*I digested chromosomal DNA with primers NSW755 and NSW756 (Table S1). PCR products were analysed by agarose gel electrophoresis and sequencing (College of Life Sciences, University of Dundee, Dundee). The positions of the Tn*5* insertions within the *S. marcescens* genome were located by BLAST analysis [41] of the genome using the publicly available server at http://www.sanger.ac.uk/resources/downloads/bacteria/serratia-marcescens.html. In order to analyse and utilise the complete sequences of relevant genes, the complete *S. marcescens* Db11 genome sequence and preliminary gene prediction were obtained from the Sanger Institute (http://www.sanger.ac.uk/resources/downloads/bacteria/serratia-marcescens.html). Db10 is the direct parent of Db11. The genomic locations of the *alb1*–*alb6* and PPTase-encoding genes are given in Table S4.

### Generalised transduction

Mini-Tn*5* insertions were introduced into a clean genetic background by generalised transduction using the φIF3 bacteriophage, according to the published procedure [42]. Transductants were selected on kanamycin and phage sensitivity was confirmed.

### RNA isolation

*S. marcescens* Db10 was spotted onto an agar plate and incubated (16 h) at 30°C. Cells were collected and processed for RNA extraction using the RiboPure™-Bacteria RNA extraction kit (Ambion®) according to the manufacturer's instructions. RNA was quantified using a Nanodrop™ spectrophotometer.

### RT-PCR

RNA was DNAse treated and cDNA was synthesised [43]. To confirm the RNA samples were free from contaminant DNA, samples of RNA lacking Superscript III (Invitrogen) were treated in parallel with samples intended for cDNA synthesis. PCR reactions using *Taq* DNA polymerase were performed according to the manufacturer's instructions (Qiagen) with 25 cycles. Primer sequences are detailed in Table S1.

### 5′ RACE

The transcription start site of *alb1* was determined using rapid amplification of cDNA ends (5′ RACE) using DNase treated RNA harvested from *S. marcescens* Db10. The method used was as described previously with the following exceptions [43]. 12.5 µl of synthesized cDNA was 5′-dA-tailed (rather than 5′-dC-tailed) by replacing the dCTP with dATP. From the poly-dA tailed cDNA, PCR was used to amplify the *alb1* promoter region using primers NSW698 and the oligo dT anchor primer (SM044) with the conditions as described previously [43]. Here a second round of PCR was necessary to increase the quantity of product using 0.625 µM oligo dT anchor primer 2 (SM045) and 0.625 µM primer AG033. The PCR product was cloned into pBluescript-KS II and ten plasmids from two independent rounds of 5′ RACE were sequenced.

### Detection of Alb4 and Alb5 from total cellular protein

To detect Alb4 and Alb5 from total cellular proteins, *S. marcescens* Db10, SAN4 (*S. marcescens* Db10 Δ*alb3*) and SAN5 (*S. marcescens* Db10 Δ*alb4–5*) were grown to stationary phase in LB (16 h) at 30°C. Cells from 0.5 ml of culture were harvested by centrifugation at 13,000 rpm for 1 min. Cells were suspended in 100 µl 2× gel sample buffer (100 mM Tris-HCl pH 6.8, 3.2% SDS, 3.2 mM EDTA, 16% glycerol, 0.2 mg/ml bromophenol blue, 2.5% β-mercaptoethanol) and boiled for 15 min. Cellular proteins were separated by SDS-PAGE using a Mini-PROTEAN TGX 4–15% Precast gradient system (Bio-rad®) and visualised by staining with Coomassie blue.

### Characterisation of the Db10 secreted compound

All strains were cultured in LB medium. Seed cultures were grown overnight at 30°C and 180 rpm. 5 ml of medium was inoculated with 10 µl of seed culture and the resulting culture was grown for 48 hours under the same conditions. Supernatant and methanol extracts of biomass were analysed by LC-MS on a Dionex 3000RS UHPLC coupled to a Bruker MaXis Q-TOF mass spectrometer. A Sigma Ascentis Express column (C18, 150×2.1 mm, 2.7 µm) was used. Mobile phases consisted of A (water containing 0.1% formic acid) and B (methanol containing 0.1% formic acid). A gradient of 20% B to 100% B in 15 minutes was employed with a flow rate of 0.2 ml/min. Absorbance at 270 nm was monitored. The mass spectrometer was operated in electrospray positive ion mode with a scan range of 50–2,000 m/z. Source conditions were: end plate offset at −500 V; capillary at −4500 V; nebulizer gas (N_2_) at 1.6 bar; dry gas (N_2_) at 8 L/min; dry Temperature at 180°C. Ion tranfer conditions: ion funnel RF at 200 Vpp; multiple RF at 200 Vpp; quadruple low mass at 55 m/z; collision energy at 5.0 ev; collision RF at 600 Vpp; ion cooler RF at 50–350 Vpp; transfer time at 121 µs; pre-Pulse storage time at 1 µs. Calibration was done with sodium formate (10 mM) through a loop injection of 20 µL of standard solution at the beginning of each run.

### Purification of althiomycin

Althiomycin was purified from the supernatant of a spent LB culture by semi-preparative HPLC (Agilent Zorbax, RP-C18, 100×21 mm, 5 µm) on an Agilent 1100 instrument. Mobile phases consisted of A: water containing 0.1% formic acid and B: methanol containing 0.1% formic acid. The flow rate was 5 ml/min and absorbance at 270 nm was monitored. A gradient of 20% B (see above) to 100% B in 25 minutes was employed for the first purification. Fractions containing althiomycin were identified by MS and pooled. The pooled fractions were freeze-dried after solvent removal at reduced pressure; the dried material was then dissolved in a small volume of methanol and re-purified on the same column with a gradient of 60% B to 100% B over 25 minutes. Fractions containing althiomycin were freeze-dried and dissolved in 200 µl of d6-DMSO for NMR analysis.

## Supporting Information

Figure S1 **Extracted ion chromatograms at** ***m/z*** **440.0600 for wild type and mutant strains of** ***S. marcescens*** **Db10.** From top to bottom: wild type, Tn mutant (NRS2992), Db10 Δ*alb2* (SAN3), Db10 Δ*alb3* (SAN4), Db10 Δ*alb4–5* (SAN5).
(TIF)

Figure S2 **Identification of a phosphopantetheinyl transferase enzyme required for althiomycin biosynthesis.** **A and B.** Sequence alignments of *S. marcescens* PPTase enzymes with the characterised PPTases used to identify them. Sequence alignments, performed using Clustal 2.1, between **A.** SMA2452 and EntD of *Escherichia coli* H730; and **B.** SMA4147 and Sfp of *Bacillus subtilis* subsp. *subtilis* RO-NN-1. **C.** Extracted ion chromatograms at *m/z* 440.0600 for wild type and mutant strains of *S. marcescens* Db10. From top to bottom: wild type, Db10 Δ*alb4–5* (SAN5), Db10 Δ*2452*::*cml* (SAN112).
(TIF)

Table S1 **Primers used in this study.**
(DOCX)

Table S2 **Bacterial strains and plasmids.**
(DOCX)

Table S3 **NMR assignment for althiomycin (DMSO-d6, 700 MHz, 25°C).**
(PDF)

Table S4 **Genomic co-ordinates for the** ***alb*** **and other relevant genes in** ***S. marcescens*** **Db11.**
(DOCX)
