# Supplementary figures and images for: The Insect Pathogen Serratia marcescens Db10 Uses a Hybrid Non-Ribosomal Peptide Synthetase-Polyketide Synthase to Produce the Antibiotic Althiomycin
Source: PLoS One. 2012 Sep 18;7(9):e44673. doi: 10.1371/journal.pone.0044673 (PMC3445576; doi:10.1371/journal.pone.0044673)

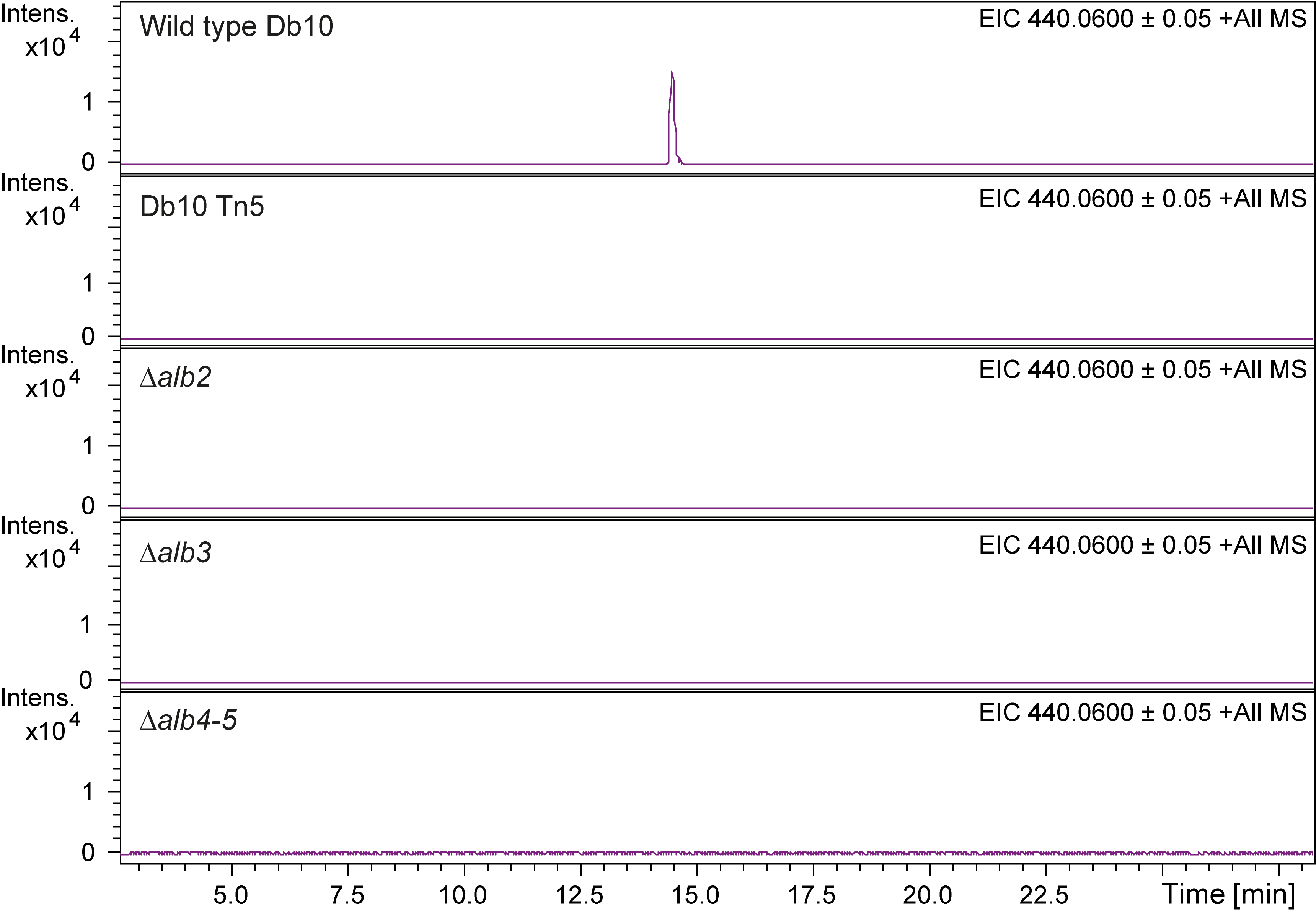

Supplement: Figure S1 — Extracted ion chromatograms at m/z 440.0600 for wild type and mutant strains of S. marcescens Db10. From top to bottom: wild type, Tn mutant (NRS2992), Db10 Δalb2 (SAN3), Db10 Δalb3 (SAN4), Db10 Δalb4–5 (SAN5). (TIF) [file pone.0044673.s001.tif]

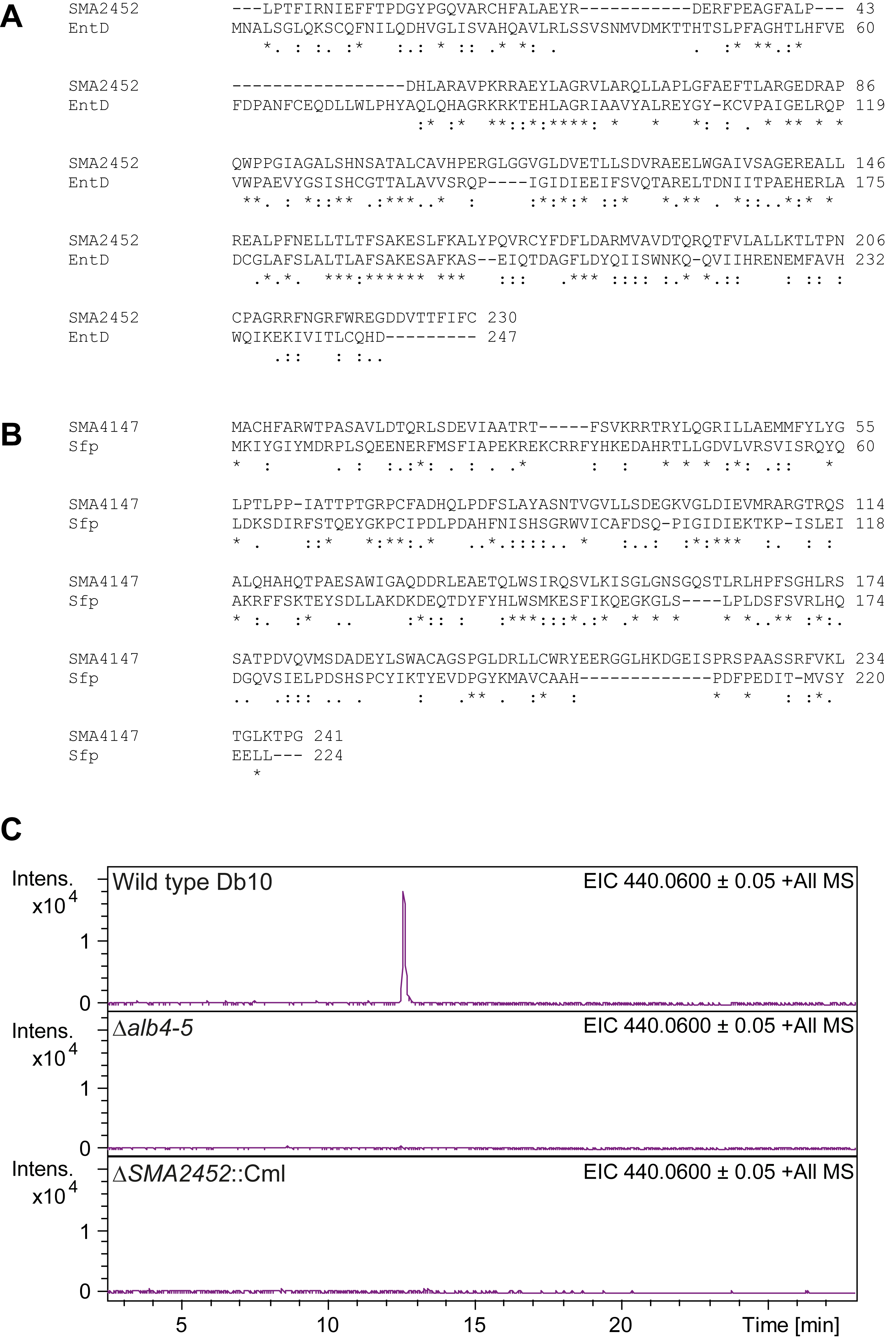

Supplement: Figure S2 — Identification of a phosphopantetheinyl transferase enzyme required for althiomycin biosynthesis. A and B. Sequence alignments of S. marcescens PPTase enzymes with the characterised PPTases used to identify them. Sequence alignments, performed using Clustal 2.1, between A. SMA2452 and EntD of Escherichia coli H730; and B. SMA4147 and Sfp of Bacillus subtilis subsp. subtilis RO-NN-1. C. Extracted ion chromatograms at m/z 440.0600 for wild type and mutant strains of S. marcescens Db10. From top to bottom: wild type, Db10 Δalb4–5 (SAN5), Db10 Δ2452::cml (SAN112). (TIF) [file pone.0044673.s002.tif]
