# Supplementary material for: The Insect Pathogen Serratia marcescens Db10 Uses a Hybrid Non-Ribosomal Peptide Synthetase-Polyketide Synthase to Produce the Antibiotic Althiomycin
Source: PLoS One. 2012 Sep 18;7(9):e44673. doi: 10.1371/journal.pone.0044673 (PMC3445576; doi:10.1371/journal.pone.0044673)
Supplement: Table S1 — Primers used in this study. (DOCX) [file pone.0044673.s003.docx]

**Supporting Table S1**

**Table S1. Primers used in this study.**

| **Primer Name** | **Sequence (5'- 3')** | **Amplified Region** |
| --- | --- | --- |
| NSW755 | GACTAGGAAGTTGAGTCG | 3’ end of the Tn5 cassette |
| NSW756 | TAGTGCGTAGAAGGGCTG | 3’ end of the Tn5 cassette |
| NSW686 | CGATAAAGACGTAGCTGGCCAT | internal *alb1* |
| NSW688 | CGTGGCGTCATTTTCAATCC | internal *alb1* |
| NSW681 | CATCAGGCGTCACTTCAAAA | internal *alb2* |
| NSW682 | TCGCCAGACATATGATCAATC | internal *alb2* |
| NSW1105 | GCATCCCGCTGGAAAACCC | internal *alb3* |
| NSW1107 | GGTGGCGTGAGGAAACTGA | internal *alb3* |
| NSW689 | GGCGCTACGTAATGCGCTTT | internal *alb4* |
| NSW674 | GGCCAAGGAAGAACAACAGGCGTT | internal *alb4* |
| NSW695 | CCAGAACTTGCTTTGATTTTCAGG | internal *alb5* |
| NSW697 | CATCTTGAGCGAGGCGAATG | internal *alb5* |
| NSW692 | GCACAACGCCTCTGAAAAG | internal *alb6* |
| NSW693 | CGGATAGCGTCATATTCTTGC | internal *alb6* |
| NSW1101 | TAACGGAGAGCGTATACCCTGC | internal *sma2294* |
| NSW1102 | CTTCGTCGAATATCAAAATGCGGAA | internal *sma2294* |
| NSW699 | GTCAAAGCTTGAAAAAACGCAC | junction *alb1* to *sma2294* |
| NSW1100 | GCAGGGTATACGCTCTCCGTTA | junction *alb1* to *sma2294* |
| NSW685 | GCGCTGTTCCCATTTATCAG | junction *alb1* to *alb2* |
| NSW687 | ATGGCCAGCTACGTCTTTATCG | junction *alb1* to *alb2* |
| NSW696 | CCTGAAAATCAAAGCAAGTTCTGG | junction *alb5* to *alb6* |
| NSW694 | GCAAGAATATGACGCTATCCG | junction *alb5* to *alb6* |
| DEN5 | TCACGRCACGAGCTGACGAC | internal 16S rRNA |
| DEN7 | ACTCCTACGGGAGGCAGC | internal 16S rRNA |
| SC2127 | TATATCTAGACAAGGCGCAGGACAAAGC | Forward primer for the upstream flanking region of *alb1*; contains *Xba*I site |
| SC2128 | TGTGAAGCTTACAGAACATTAATCCGAATGTTAATAACC | Reverse primer for the upstream flanking region of *alb1*; contains *HinD*III site |
| SC2129 | TATAAAGCTTCCCGGGAAAGCTGAAGAATAAATAATCGTTTTCGC | Forward primer for the downstream flanking region of *alb1*; contains *HinD*III site |
| SC2130 | TATAGGGCCCAGAACTCCACCAGCTCTCTCACC | Reverse primer for the downstream flanking region of *alb1*; contains *Apa*I site |
| SC2155 | TGTGTCTAGAATTCATGCGATTATCGTTGATTCC | Forward primer for the upstream flanking region of *alb2*; contains *Xba*I site |
| SC2156 | TGTGAAGCTTATTTTCCATAGATATTCTCCAGATGAAGG | Reverse primer for the upstream flanking region of *alb2*; contains *HinD*III site |
| SC2157 | TATAAAGCTTCCCGGGCAGGGATTCGAAAATATTGGTTGG | Forward primer for the downstream flanking region of *alb2*; contains *HinD*III site |
| SC2158 | TATAGGGCCCGACGATACAAAGCCACC | Reverse primer for the downstream flanking region of *alb2*; contains *Apa*I site |
| SC2151 | TATATCTAGACAGATTAAATTGAATGCCTATACCGTC | Forward primer for the upstream flanking region of *alb3*; contains *Xba*I site |
| SC2152 | TATAAAGCTTGAAACTATCCATACCCATCTCCATGA | Reverse primer for the upstream flanking region of *alb3*; contains *HinD*III site |
| SC2153 | TATAAAGCTTCCCGGGGCGGTAAAACCCTGATAACCC | Forward primer for the downstream flanking region of *alb3*; contains *HinD*III site |
| SC2154 | TATAGGGCCCTGGAAAGCCAACAGCAAAGC | Reverse primer for the downstream flanking region of *alb3*; contains *Apa*I site |
| SC2123 | TATATCTAGATACTACCGTTATCAGACCTTGCTGG | Forward primer for the upstream flanking region of *alb4*; contains *Xba*I site |
| SC2124 | TGTGAAGCTTAATCTTCATGGATATTACTCTTTTGGG | Reverse primer for the upstream flanking region of *alb4*; contains *HinD*III site |
| SC2125 | TATAAAGCTTCCCGGGTGGACGCTGGCTTGATGG | Forward primer for the downstream flanking region of *alb5*; contains *HinD*III site |
| SC2126 | TATAGGGCCCGCTTCTGAAATCAACGTCATCC | Reverse primer for the downstream flanking region of *alb5*; contains *Apa*I site |
| AG011 | TATAAAGCTTCTCAAACCACATACGG  CTTCC | Forward primer for the upstream flanking region of *alb6*; contains *HinD*III site |
| AG012 | TATATCTAGAAGGACAGGGAAATCT  GCC | Reverse primer for the upstream flanking region of *alb6*; contains *Xba*I site |
| AG013 | TATAAAGCTTCAGCAGCTGGCGTAAA  GCG | Forward primer for the downstream flanking region of *alb6*; contains *HinD*III site |
| AG014 | TATAGGGCCCACCTTTCTGGCGGCGG  AATAC | Reverse primer for the downstream flanking region of *alb6*; contains *Apa*I site |
| AG063 | TATATCTAGAGCGAACCTTGTACCGTGAG | Forward primer for the upstream flanking region of *SMA4147*; contains *Xba*I site |
| AG064 | TATAAAGCTTAAAATGACACGCCATTG | Reverse primer for the upstream flanking region of *SMA4147*; contains *HinD*III site |
| AG065 | TATAAAGCTTAAGACGCCCGGTTAAC | Forward primer for the downstream flanking region of *SMA4147*; contains *HinD*III site |
| AG067 | TATATCTAGACTATCAGGATTCGCTGTTCAC | Forward primer for the upstream flanking region of *SMA2452*; contains *Xba*I site |
| AG068 | TATAAAGCTTGATAAAAGTGGGCAACG | Reverse primer for the upstream flanking region of *SMA2452*; contains *HinD*III site |
| AG071 | TATAGGGCCCTTGTTTAAAGCGGCTGAC | Reverse primer for the downstream flanking region of *SMA2452*; contains *Apa*I site |
| AG072 | TATAAAGCTTGAAGGTGACGATGTCACG | Forward primer for the downstream flanking region of *SMA2452*; contains *HinD*III site |
| AG049 | TATAGGGCCCGAATATGAAAATGCG | Forward primer for the upstream flanking region of the *alb* promoter; contains *Apa*I site |
| AG050 | TATAAAGCTTCCCTCTTCTGTCATGAGC | Reverse primer for the upstream flanking region of the *alb* promoter; contains *HinD*III site |
| AG051 | TATAAAGCTTAAATCATAAAAAATTTATTTGCTTTG | Forward primer to amplify the T5 promoter from pQE80; contains *HinD*III site |
| AG052 | TATAGGATCCTGTGTGAAATTGTTATCCG | Reverse primer to amplify the T5 promoter from pQE80; contains *Bam*HI site |
| AG053 | TATAGGATCCTTAACATTCGGATTAATGTTCTG | Forward primer for the downstream flanking region of the *alb* promoter; contains *Bam*HI site |
| AG054 | TATATCTAGATTTCTTCGTTTCCGGCAG | Reverse primer for the downstream flanking region of the *alb* promoter; contains *Xba*I site |
| AG001 | GCATGGATCCATGTTCTGTTGGAG | Forward primer for the *alb1* coding region; contains *Bam*HI site for cloning into pSUPROM |
| AG002 | GCATCTCGAGTTATTCTTCAGCTTTGC | Reverse primer for the *alb1* coding region; contains *Xho*I site for cloning into pSUPROM |
| AG003 | GCATGGATCCATGGAAAATGAAACC | Forward primer for the *alb2* coding region; contains *Bam*HI site for cloning into pSUPROM |
| AG004 | GCATCTCGAGTTAAATTATCCAACC | Reverse primer for the *alb2* coding region; contains *Xho*I site for cloning into pSUPROM |
| AG007 | GCATGGATCCATGGATAGTTTCGCCT  CGCAT | Forward primer for the *alb3* coding region; contains *Bam*HI site for cloning into pSUPROM |
| AG008 | GCATCTCGAGTCAGGGTTTTACCGCC  ACGAG | Reverse primer for the *alb3* coding region; contains *Xho*I site for cloning into pSUPROM |
| AG035 | TATAGGATCCTTGATTACGCCGCTCCCG | Forward primer for the *alb6* coding region; contains *Bam*HI site for cloning into pSUPROM |
| AG036 | GATACTCGAGTTACGCCAGCTGCTGATTC | Reverse primer for the *alb6* coding region; contains *Xho*I site for cloning into pSUPROM |

^a^ Primer engineered DNA restriction sites are underlined.
