# Supplementary material for: The Insect Pathogen Serratia marcescens Db10 Uses a Hybrid Non-Ribosomal Peptide Synthetase-Polyketide Synthase to Produce the Antibiotic Althiomycin
Source: PLoS One. 2012 Sep 18;7(9):e44673. doi: 10.1371/journal.pone.0044673 (PMC3445576; doi:10.1371/journal.pone.0044673)
Supplement: Table S2 — Bacterial strains and plasmids. (DOCX) [file pone.0044673.s004.docx]

**Supporting table S2**

**Table S2. Bacterial Strains and Plasmids**

| **Bacterial Strain / Plasmid** | **Description ^a^** | **Source or Reference ^b^** |
| --- | --- | --- |
| *Bacillus subtilis* |  |  |
| NCIB3610 | Wild type prototroph | B.G.S.C. |
| NRS1473 | NCIB3610 *sacA-P* hy-spac*-gfp* (Kan) | [1] |
|  |  |  |
| *Micrococcus luteus* ATCC4698 | ATCC4698 | M. Bibb |
|  |  |  |
| *Staphylococcus aureus* 113 | ATCC35556 | T. Palmer |
|  |  |  |
| *Escherichia coli* |  |  |
| MC1061 | *F'lacIQ lacZM15* Tn*10* (Tet) Cloning host | [2] |
| CC118λ*pir* | Cloning host and donor strain for pKNG101-derived marker exchange plasmids (λ*pir*) | [3] |
| HH26 pNJ5000 | Mobilizing strain for conjugal transfer | [4] |
|  |  |  |
| *Serratia marcescens* |  |  |
| Db10 | Wild type strain | [5] |
| NRS2992 | *S. marcescens* Db10 *SMA2290::*Tn*5* | This Study |
| SJC13 | *S. marcescens* Db10 *SMA2290::*Tn*5* | This Study |
| SAN2 | *S. marcescens* Db10 (Δ*alb1*) in-frame | This study |
| SAN3 | *S. marcescens* Db10 (Δ*alb2*) in-frame | This study |
| SAN4 | *S. marcescens* Db10 (Δ*alb3*) in-frame | This study |
| SAN88 | *S. marcescens* Db10 (Δ*alb3_51-262_*) in-frame | This study |
| SAN5 | *S. marcescens* Db10 (Δ*alb4-5*) in-frame | This study |
| SAN60 | *S. marcescens* Db10 (Δ*alb6*) in-frame | This study |
| SAN96 | *S. marcescens* Db10 (Δ*SMA4147*) in-frame | This study |
| SAN112 | *S. marcescens* Db10 (Δ*SMA2452*::Cml) | This study |
| SAN100 | *S. marcescens* Db10 P_T5_-*alb1-6* (T5 promoter replacing native promoter upstream of *alb1*) | This study |
| ATCC274 | Wild type | A.T.C.C. |
|  |  |  |
| **Plasmids** |  |  |
| pBluescript KS+ | High copy cloning vector (Ap^R^) | Stratagene |
| pKNG101 | Suicide vector for marker exchange (Sm^R^, *sacBR*, *mobRK2,* ori *R6K*) | [6] |
| pQE-80L | Protein overexpression vector; source of T5 promoter | Qiagen |
| pSUPROM | Vector for constitutive expression of cloned genes under the control of the *E. coli* Tat promoter (Kan^R^) | [7] |
| pNW573 | pKNG101-derived marker exchange plasmid for the generation of chromosomal Δ*alb1* | This study |
| pNW577 | pKNG101-derived marker exchange plasmid for the generation of chromosomal Δ*alb2* | This study |
| pNW579 | pKNG101-derived marker exchange plasmid for the generation of chromosomal Δ*alb3* | This study |
| pSAN24 | pKNG101-derived marker exchange plasmid for the generation of chromosomal Δ*alb3* (51-262) | This study |
| pNW572 | pKNG101-derived marker exchange plasmid for the generation of chromosomal Δ*alb4-5* | This study |
| pSAN8 | pKNG101-derived marker exchange plasmid for the generation of chromosomal Δ*alb6* | This study |
| pSAN31 | pKNG101-derived marker exchange plasmid for the generation of chromosomal Δ*SMA4147* | This study |
| pSAN40 | pKNG101-derived marker exchange plasmid for the generation of chromosomal Δ*SMA2452*::Cml | This study |
| pSAN33 | pKNG101-derived marker exchange plasmid for introducing the T5 promoter upstream from *alb1* | This study |
| pSAN1 | *alb1* coding sequence in pSUPROM | This study |
| pSAN2 | *alb2* coding sequence in pSUPROM | This study |
| pSAN3 | *alb3* coding sequence in pSUPROM | This study |
| pSAN38 | *alb6* coding sequence in pSUPROM | This study |
| pSAN46 | *SMA2452* coding sequence in pSUPROM | This study |

^a.^ The antibiotic resistances are represented as follows: Ap, ampicillin; Cml, chloramphenicol; Kan, kanamycin; Tet, tetracycline; Sm, streptomycin.

^b.^ B.G.S.C. is the *Bacillus* genetic stock centre.

**References**

1. Verhamme DT, Kiley TB, Stanley-Wall NR (2007) DegU co-ordinates multicellular behaviour exhibited by *Bacillus subtilis*. Mol Microbiol 65: 554-568.

2. Perego M, Hoch JA (1988) Sequence analysis and regulation of the *hpr* locus, a regulatory gene for protease production and sporulation in *Bacillus subtilis*. J Bacteriol 170: 2560-2567.

3. Herrero M, de Lorenzo V, Timmis KN (1990) Transposon vectors containing non-antibiotic resistance selection markers for cloning and stable chromosomal insertion of foreign genes in Gram-negative bacteria. J Bacteriol 172: 6557-6567.

4. Grinter NJ (1983) A broad-host-range cloning vector transposable to various replicons. Gene 21: 133-143.

5. Flyg C, Kenne K, Boman HG (1980) Insect pathogenic properties of *Serratia marcescens*: phage-resistant mutants with a decreased resistance to *Cecropia* immunity and a decreased virulence to *Drosophila*. J Gen Microbiol 120: 173-181.

6. Kaniga K, Delor I, Cornelis GR (1991) A wide-host-range suicide vector for improving reverse genetics in gram-negative bacteria: inactivation of the *blaA* gene of *Yersinia enterocolitica*. Gene 109: 137-141.

7. Jack RL, Buchanan G, Dubini A, Hatzixanthis K, Palmer T, et al. (2004) Coordinating assembly and export of complex bacterial proteins. EMBO J 23: 3962-3972.
