# Supplementary material for: The Insect Pathogen Serratia marcescens Db10 Uses a Hybrid Non-Ribosomal Peptide Synthetase-Polyketide Synthase to Produce the Antibiotic Althiomycin
Source: PLoS One. 2012 Sep 18;7(9):e44673. doi: 10.1371/journal.pone.0044673 (PMC3445576; doi:10.1371/journal.pone.0044673)
Supplement: Table S3 — NMR assignment for althiomycin (DMSO-d6, 700 MHz, 25°C). (PDF) [file pone.0044673.s005.pdf]

**Supporting Table S3**

**Table S3. NMR assignment for althiomycin (DMSO-d6, 700MHz, 25°C)**

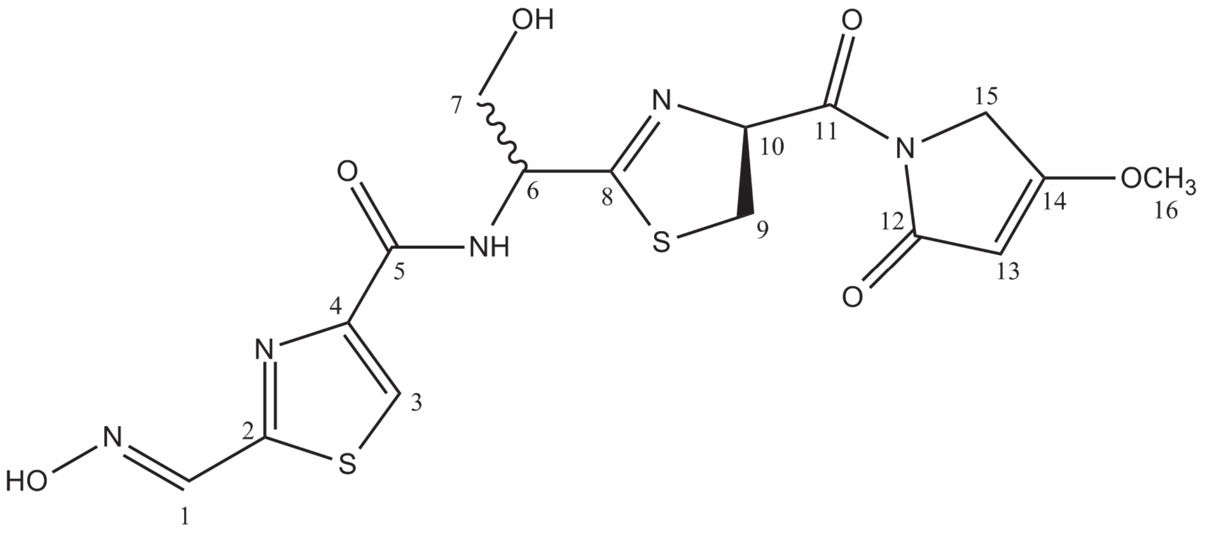


| Carbon | $\delta_H$(ppm) | $\delta_C$ (ppm)* | HMBC | Literature** | |
|---|---|---|---|---|---|
| | | | | $\delta_H$(ppm) | $\delta_C$ (ppm) |
| 1 | 8.37(s) | 143.0 | C2 | **8.37(s)** | **143.2** |
| 2 | | 162.6 | | | **162.6** |
| 3 | 8.29 (s) | 124.8 | C2, C5 | **8.35(s)** | **125.3** |
| 4 | | 149.6 | | | **149.7** |
| 5 | | 160.4 | | | **159.8** |
| C5-NH-C6 | 8.39(br) | | C5, C6 | **8.41(m)** | |
| 6 | 4.84(m) | 53.5 | C5, C7, C8 | **4.88(m)** | **53.9** |
| 7 | 3.79(m) | 61.8 | C6, C8 | **3.80(m)** | **62.2** |
| 8 | | 173.5 | | | **173.4** |
| 9 | 3.40/3.58(m) | 34.9 | C8, C10, C11 | **3.40/3.58(m)** | **34.6** |
| 10 | 6.14(t, 8.6) | 77.8 | C8, C9, C11 | **6.16(m)** | **78.0** |
| 11 | | 168.1 | | | **168.3** |
| 12 | | 169.5 | | | **169.6** |
| 13 | 5.39(s) | 94.3 | C12, C15, C16 | **5.43(m)** | **94.4** |
| 14 | | 177.2 | | | **177.2** |
| 15 | 4.29(s) | 47.9 | C11, C12, C13, C14 | **4.33(m)** | **48.0** |
| 16 | 3.88(s) | 58.9 | C14 | **3.88(s)** | **59.2** |

* Carbon shift obtained from HSQC and HMBC

**Reference:

Cortina NS, Revermann O, Krug D, Muller R (2011) Identification and characterization of the althiomycin biosynthetic gene cluster in *Myxococcus xanthus* DK897. Chembiochem 12: 1411-1416.
