# Supplementary material for: The Insect Pathogen Serratia marcescens Db10 Uses a Hybrid Non-Ribosomal Peptide Synthetase-Polyketide Synthase to Produce the Antibiotic Althiomycin
Source: PLoS One. 2012 Sep 18;7(9):e44673. doi: 10.1371/journal.pone.0044673 (PMC3445576; doi:10.1371/journal.pone.0044673)
Supplement: Table S4 — Genomic co-ordinates for the alb and other relevant genes in S. marcescens Db11. (DOCX) [file pone.0044673.s006.docx]

**Supporting table S4**

**Table S4. Genomic co-ordinates for the *alb* and other relevant genes in *S. marcescens* Db11.**

| **CDS** | **Name** | **Genomic location** |
| --- | --- | --- |
| *SMA2293* | *alb1* | complement(2441007..2442188) |
| *SMA2292* | *alb2* | complement(2439952..2440908) |
| *SMA2291* | *alb3* | complement(2439026..2439937) |
| *SMA2290* | *alb4* | complement(2431874..2439004) |
| *SMA2289* | *alb5* | complement(2415828..2431871) |
| *SMA2288* | *alb6* | complement(2414947..2415681) |
| *SMA2294* |  | complement(2442494..2443843) |
| *SMA2452* |  | complement(2594348..2595040) |
| *SMA4147* |  | complement(4428375..4429100) |

The complete genome sequence is available at: http://www.sanger.ac.uk/resources/downloads/bacteria/serratia-marcescens.html
